# Barriers from calling ambulance after recognizing stroke differed in adults younger or older than 75 years old in China

**DOI:** 10.1186/s12883-019-1480-6

**Published:** 2019-11-12

**Authors:** Shengde Li, Li-Ying Cui, Craig Anderson, Chunpeng Gao, Chengdong Yu, Guangliang Shan, Longde Wang, Bin Peng, on behalf of the FAST-RIGHT Investigators and Coordinators, Baohua Chao, Lei Cao, Lingxiao Wang

**Affiliations:** 1 Department of Neurology, Peking Union Medical College Hospital, Peking Union Medical College and Chinese Academy of Medical Sciences, Shuaifuyuan1, Dong Cheng District, Beijing, 100730 China; 2 Neurological and Mental Health Division, The George Institute for Global Health, Faculty of Medicine, University of New South Wales, Sydney, Australia; 3 The George Institute for Global Health, Peking University Health Science Center, Beijing, China; 4 Disease Control and Prevention Office, Dalian Municipal Central Hospital, Liaoning, China; 5 Department of Epidemiology and Statistics, Institute of Basic Medical Sciences, Chinese Academy of Medical Sciences, Beijing, China; 6 Stroke Control Project Committee, The National Health Commission, Beijing, China

**Keywords:** Stroke, Emergency medical services, Aging, Awareness, Risk factors, Healthy behaviors, Family, Health education

## Abstract

**Background:**

As health behavior varies with increasing age, we aimed to examine the potential barriers in calling emergency medical services (EMS) after recognizing a stroke among 40–74- and 75–99-year-old adults.

**Methods:**

Data were obtained from a cross-sectional community-based study (FAST-RIGHT) that was conducted from January 2017 to May 2017 and involved adults (age ≥ 40 years) across 69 administrative areas in China. A subgroup of residents (153675) who recognized stroke symptoms was analyzed. Multivariable logistic regression models were performed in the 40–74 and 75–99 age groups, separately, to determine the factors associated with wait-and-see behaviors at the onset of a stroke.

**Results:**

In the 40–74 and 75–99 age groups, the rates of participants who chose “Self-observation at home” were 3.0% (3912) and 3.5% (738), respectively; the rates of “Wait for family, then go to hospital” were 31.7% (42071) and 33.1% (6957), respectively. Rural residence, living with one’s spouse, low income (< 731 US $ per annum), having a single avenue to learn about stroke, and having friends with stroke were factors associated with waiting for one’s family in both groups. However, unlike in the 40–74 age group, sex, number of children, family history, and stroke history did not influence the behaviors at stroke onset in the 75–99 age group.

**Conclusions:**

Different barriers from recognizing stroke and calling an ambulance exist in the 40–74 and 75–99 age groups in this specific population. Different strategies that mainly focus on changing the “Wait for family” behavior and emphasize on immediately calling EMS are recommended for both age groups.

## Introduction

Stroke has become the leading cause of death and disability in China with its incidence increasing in recent times [1, 2]. Thrombolysis with intravenous alteplase reduces disability associated with acute ischemic stroke and improves patient outcomes. However, most patients fail to reach the hospital in time to qualify for reperfusion therapy [3, 4]. The main reason for this is the delayed arrival to the emergency department, with less than 25% of the patients arriving within 3 h [3, 4]. Evidence indicates that use of emergency medical services (EMS) use is crucial to reduce pre-hospital delay [4–7]. However, the use of EMS at the time of stroke involves a complex “knowledge-to-action” process [8, 9]. Although recognition of stroke symptoms may prompt the intent to call EMS [10, 11], knowledge of stroke does not necessarily imply that the individual will call EMS [9, 12, 13]. Thus, only some of the individuals who recognize the onset of a stroke will call an ambulance [9, 10, 12–14], indicating a huge gap between the knowledge of stroke symptoms and appropriate action [6]. Our previous report from FAST-RIGHT also showed that 34.9% of adults recognized stroke onset but failed to call EMS [15]. Adults in the 75–99 age group are likely to experience stroke onset more frequently than those in the 40–74 age group [16]. However, greater disability and cognitive impairment, and the poor use of health care in the older population may negatively affect their behavior towards stroke onset [17, 18]. The potential factors behind wait-and-see behaviors may be different in the 75–99 age group. Therefore, based on data collected from the FAST-RIGHT study, we aimed to determine the risk factors associated with avoiding an immediate ambulance call when identifying stroke onset, and whether these factors differed between the 40–74 and 75–99 age groups.

## Methods

The data were derived from the FAST-RIGHT database, which is part of the China National Stroke Screening Survey (CNSSS). More details of the CNSSS can be found on the CNSSS official website [19], and have also been described in our previous publications [15, 20]. Briefly, the CNSSS was a cross-sectional community-based survey with a 2-stage stratified sampling framework based on county-level demographic data that was conducted between January 2017 and May 2017. The FAST-RIGHT study recruited 69 of the 221 administrative areas from the CNSSS and only screened residents aged 40 years and above in each community [15]. All participants were screened by trained research staff using a standard face-to-face questionnaire that covered information about socio-demographic, medical and family history, lifestyle factors, and included four specific questions regarding stroke awareness (See Additional file 1: Appendix 2). All screening data were transferred from questionnaires to an electronic database and checked centrally for completeness and errors by an experienced data manager. Finally, individuals who recognized stroke symptoms in the FAST-RIGHT study were analyzed for our report. The FAST-RIGHT study was approved by the central ethics committee of Peking Union Medical College Hospital (the principal study center), and all participants provided written informed consent.

### Explanatory and outcome variables

We defined recognition of stroke symptoms as a participant’s unprovoked awareness of “facial droop,” “arm weakness,” and “speech disturbances (slurred speech or word-finding difficulties)” [21]. Calling EMS immediately after the onset of any of these symptoms was regarded as the correct action in response to stroke. Choosing “Self-observation at home” and “Call and wait for family member, and then go to hospital (Wait for family)” at the time of stroke were defined as detrimental “wait-and-see” behaviors. A reported history of stroke was confirmed by a neurologist or physician, who applied standard diagnostic criteria with any available brain neuroimaging data. The cardiovascular risk factors were hypertension, diabetes mellitus, dyslipidemia, atrial fibrillation (AF)/valvular heart disease, overweight/obesity, smoking, physical inactivity, and family history of stroke, which are based on standard definitions (See Additional file 1: Appendix 3). In terms of age, the participants were divided into the 40–74 age group and the 75–99 age group [16].

### Statistical analysis

All participants who recognized their stroke symptoms in the FAST-RIGHT study were included in the analysis. We performed descriptive analysis for socio-demographic data and some other related variables. Pearson’s chi-squared tests were conducted to compare the two types of detrimental wait-and-see behaviors between the subgroups. Multivariable logistic regression analysis was performed to identify the factors associated with different wait-and-see behaviors after stroke recognition in the different age groups, and in the total study population. Adjusted odds ratios (ORs) and 95% confidence intervals (CIs) for each variable were calculated. All analyses were performed using SAS version 9.3, and a standard 2-sided *P* value (*P* < 0.05) was considered statistically significant.

## Results

Out of the 187,723 respondents in the FAST-RIGHT database, 153,675 recognized the symptoms of stroke and were included in our final analysis with limited missing data (See Additional file 1: Table S1 and Figure S1). We also compared the sex and county sites between the different age groups. We found the higher age group (75–99 years old) with higher proportions of low education (65.5% vs 38.8%), low income (38.6% vs 24.3%), multiple children (91.1% vs 67.5%) and the single avenue to learn about stroke (47.2% vs 46.6%) were higher in 75–99 age group (See Additional file 1: Table S2). Avenues to gaining knowledge of stroke included higher usage of newspaper (30.0% vs 26.4%) and lower utilization of internet (3.5% vs. 8.4%) in 75–99 age group. However, we found that the usage of television, broadcast, and popular science and technology activity were similar between two groups. The most popular avenue was television (See Additional file 1: Table S3). The prevalence rate of stroke were 3.1% (4175/132587) in 40–49 age group, and 5.6% (1173/20983) for the 75–99 age group (See Additional file 1: Figure S2).

The rates of individuals choosing “self-observation at home” and “wait for family” option were 3.0% (*n* = 3912) and 31.7% (*n* = 42,071) in the 40–74 age group, and 3.5% (*n* = 738) and 33.1% (*n* = 6957) in the 75–99 age group, respectively (See Additional file 1: Table S4). The differences in the wait-and-see behaviors in both age groups are shown in Table 1. The rates of inappropriate responses to stroke varied across the socio-demographic subgroups, but the most frequent response was to “wait for family, then go to hospital.” Out of the total population, the 75–99 age group were less likely to stay at home for self-observation (OR, 0.88; 95% CI, 0.81–0.97) and wait for their family (OR, 0.88; 95% CI, 0.85–0.91) (See Additional file 1: Table S5).

**Table 1:** Baseline characteristics and responses to stroke among residents recognizing stroke by demographic, and socio-economic variables

	40–74 age group Responses to stroke, N (%)			75–99 age group Responses to stroke, N (%)		
Variables	Self-observation at home	Wait for family	Call EMS	Self-observation at home	Wait for family	Call EMS
Sex						
Male	1858 (3.1)	19,424 (32.2)	38,988 (64.7)	313 (3.2)	3237 (33.4)	6141 (63.4)
Female	2054 (2.8)	22,647 (31.3)	47,688 (65.9)	425 (3.8)	3720 (32.9)	7156 (63.3)
Site						
Urban	2301 (3.4)	15,835 (23.2)	50,155 (73.4)	360 (3.4)	2909 (27.3)	7401 (69.3)
Rural	1611 (2.5)	26,236 (40.8)	36,521 (56.7)	378 (3.7)	4048 (39.2)	5896 (57.1)
Regions						
North + Northeast	143 (1.4)	3075 (30.1)	6991 (68.5)	16 (1.7)	291 (31.0)	633 (67.3)
East	1136 (2.7)	12,447 (30.1)	27,800 (67.2)	193 (3.5)	1796 (32.2)	3585 (64.2)
Central	1797 (4.4)	12,645 (30.6)	26,842 (65.0)	354 (4.9)	2346 (32.2)	4582 (62.9)
South	429 (3.2)	5108 (37.6)	8039 (59.2)	72 (2.1)	1019 (29.3)	2382 (68.6)
Southwest	254 (1.7)	3712 (25.3)	10,698 (73.0)	64 (3.0)	687 (31.9)	1404 (65.1)
Northwest	153 (1.3)	5084 (44.1)	6306 (54.6)	39 (2.5)	818 (52.2)	711 (45.3)
Education						
≤ Primary	1286 (2.5)	21,065 (40.9)	29,192 (56.6)	426 (3.1)	4802 (34.9)	8527 (62.0)
Middle/High school	2361 (3.3)	19,060 (26.5)	50,583 (70.2)	288 (4.6)	1842 (29.4)	4144 (66.0)
≥ College	265 (2.9)	1945 (21.4)	6900 (75.7)	24 (2.5)	312 (32.5)	624 (65.0)
Annual Income^a^, US $						
< 731	911 (2.8)	14,958 (46.5)	16,314 (50.7)	253 (3.1)	3252 (40.2)	4588 (56.7)
731–2923	1322 (2.9)	15,076 (32.7)	29,705 (64.4)	229 (3.7)	2013 (32.1)	4019 (64.2)
> 2923	1679 (3.1)	12,001 (22.1)	40,653 (74.8)	256 (3.8)	1688 (25.5)	4685 (70.7)
Living Status^b^						
Alone	87 (3.0)	772 (26.3)	2075 (70.7)	38 (2.7)	424 (29.8)	960 (67.5)
With spouse	3501 (2.9)	38,976 (31.8)	79,941 (65.3)	569 (3.5)	5414 (33.5)	10,175 (63.0)
With others	317 (4.6)	2241 (32.6)	4326 (62.8)	128 (3.8)	1104 (33.0)	2111 (63.2)
Children number						
0	29 (2.6)	300 (26.9)	785 (70.5)	3 (2.7)	36 (32.1)	73 (65.2)
1	1376 (3.3)	8712 (20.8)	31,818 (75.9)	115 (6.6)	541 (30.9)	1096 (62.5)
2–3	1887 (2.4)	28,847 (36.4)	48,514 (61.2)	388 (3.1)	3969 (31.8)	8110 (65.1)
≥ 4	619 (6.1)	4176 (41.0)	5383 (52.9)	232 (3.5)	2407 (36.3)	3999 (60.2)
Stroke in others ^c^						
No	2787 (2.6)	34,582 (32.4)	69,340 (65.0)	513 (3.1)	5545 (33.0)	10,729 (63.9)
Yes	1125 (4.3)	7488 (28.9)	17,336 (66.8)	225 (5.3)	1412 (33.6)	2568 (61.1)
Avenues^d^						
1	1790 (2.9)	24,059 (38.9)	35,987 (58.2)	355 (3.6)	3942 (39.8)	5605 (56.6)
2–3	2034 (3.2)	16,518 (26.3)	44,341 (70.5)	374 (3.6)	2817 (27.1)	7203 (69.3)
4–6	88 (1.1)	1471 (18.6)	6346 (80.3)	9 (1.3)	194 (28.0)	489 (70.7)
Family history of stroke						
No	3127 (2.7)	36,348 (31.3)	76,533 (66.0)	562 (3.1)	5975 (33.0)	11,595 (63.9)
Yes	183 (2.0)	2501 (26.5)	6739 (71.5)	23 (2.3)	340 (34.4)	625 (63.3)
Unknown	600 (8.5)	3159 (44.4)	3350 (47.1)	153 (8.2)	636 (34.2)	1072 (57.6)
History of CVD^e^						
No	3839 (3.0)	40,812 (31.8)	83,761 (65.2)	714 (3.6)	6563 (33.1)	12,533 (63.3)
Yes	71 (1.7)	1229 (29.4)	2875 (68.9)	24 (2.1)	391 (33.3)	758 (64.6)

^a^Personal annual income

^b^With spouse includes living with spouse or both spouse and children; With others includes living with children, living in nursing home, and with other people. Those with other type of living status were classified as missing data

^c^Relatives or colleagues who have suffered an acute stroke

^d^Number of avenues taken to learn about acute stroke

^e^CVD denotes cerebral vascular disease, including ischemic stroke, transient ischemic anemia, cerebral hemorrhage, and subarachnoid hemorrhage

The rate of living alone increased from 1.3% in 40–49 year–old group to 7.7% in 80–99 year–old group (See Additional file 1: Table S6). However, about 30% did not intend to call EMS. Living alone showed low likelihood to wait for family (OR, 0.75; 95% CI, 0.70–0.80), but was not related to rejecting self-observation at home (See Additional file 1: Table S5). In the 75–99 age group, the absolute rate of waiting for family in those living alone was lower by 3.7%, compared with those living with a spouse.

**Fig. 1:**
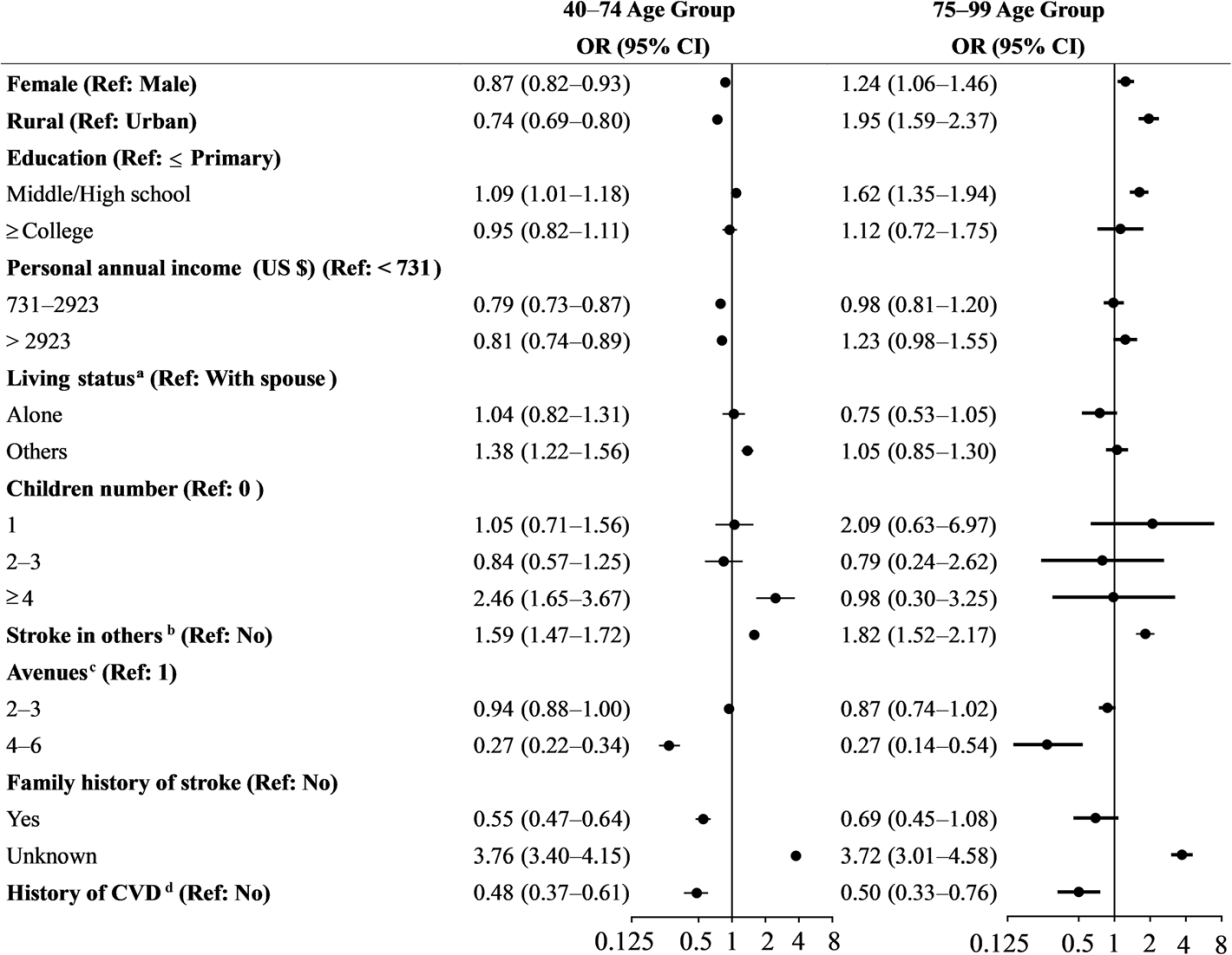
Logistic regression models of factors associated with self-observation at home among residents recognizing stroke. This logistic model was adjusted by regions. ^a^ With spouse includes living with spouse or both spouse and children; With others includes living with children, living in nursing home, and with other people. Those with other type of living status were classified as missing data. ^b^ Relatives or colleagues who have suffered an acute stroke. ^c^ Number of avenues taken to learn about acute stroke. ^d^ CVD denotes cerebral vascular disease, including ischemic stroke, transient ischemic anemia, cerebral hemorrhage, and subarachnoid hemorrhage

The rates of staying at home for self–observation varied from 1.1–8.5% across the different subgroups in the 40–74 age group, and 1.3–8.2% in the 75–99 age group. The factors associated with “Self-observation at home” in the 40–74 age group, and the 75–99 age group are shown in Fig. 1. Contrary to the 40–74 age group, income level and family history of stroke were not associated with staying at home in the 75–99 age group. Females younger than 75 years were less likely to wait for self–observation at home, but those over 75 years prefer self-observation at home (OR, 1.24; 95% CI: 1.06–1.46). Compared with urban area, rural individuals’ odds of choosing “Self-observation at home” decreased by 26% in the 40–74 age group, while increased by 95% in the 75–99 age group. Those having relatives or colleagues afflicted by stroke were 59% more likely to stay at home for self-observation in the 40–74 age group, compared to 82% in the 75–99 age group.

**Fig. 2:**
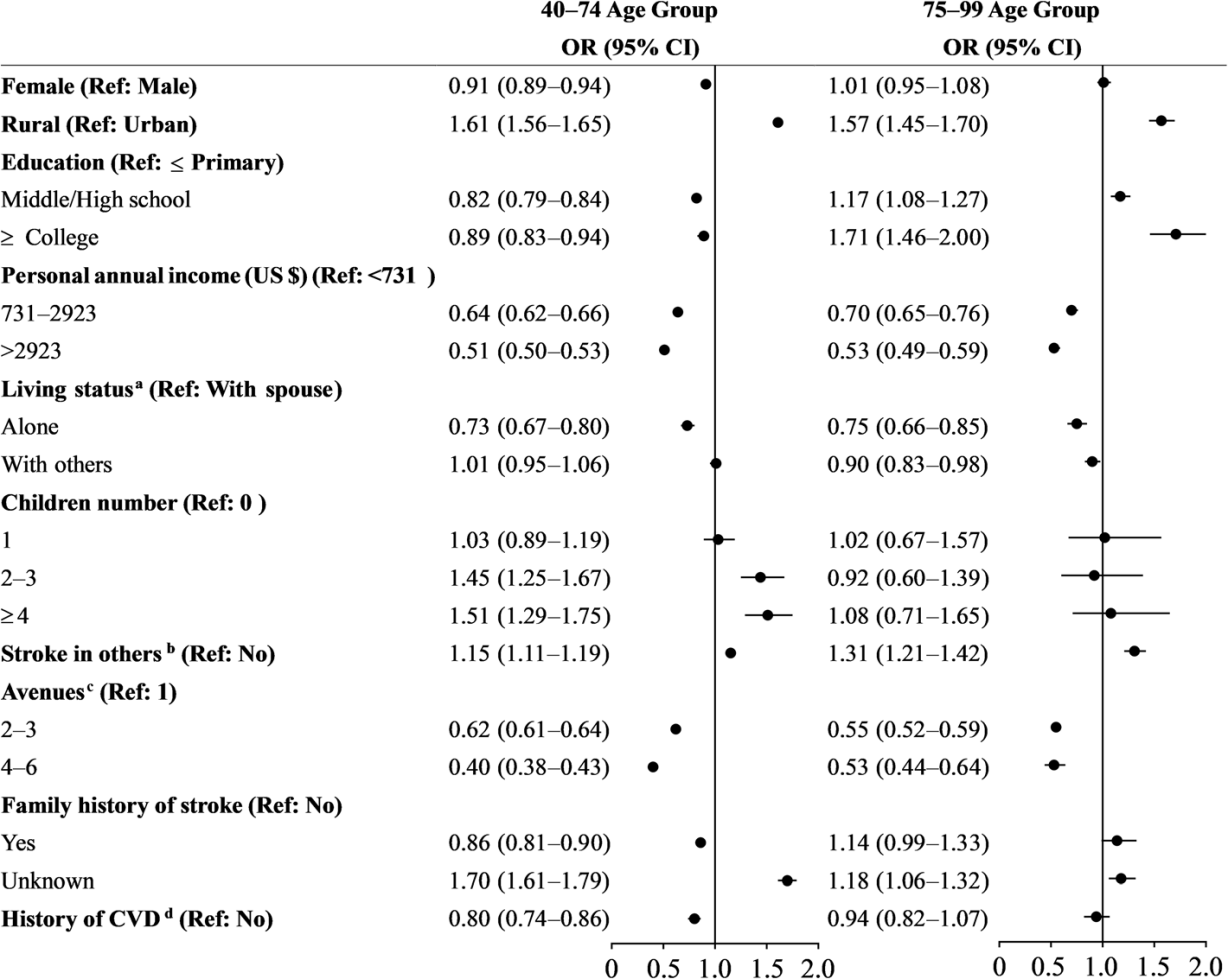
Logistic regression models of factors associated with waiting for family among residents recognizing stroke. This logistic model was adjusted by regions. ^a^ With spouse includes living with spouse or both spouse and children; With others includes living with children, living in nursing home, and with other people. Those with other type of living status were classified as missing data. ^b^ Relatives or colleagues who have suffered an acute stroke. ^c^ Number of avenues taken to learn about acute stroke. ^d^ CVD denotes cerebral vascular disease, including ischemic stroke, transient ischemic anemia, cerebral hemorrhage, and subarachnoid hemorrhage

Figure 2 presents the associated factors with waiting for family. In the 40–74 age group, living in a rural location, and having multiple children indicated waiting for the family, while these factors did not affect the intents of individuals in the 75–99 age group. Similarly, females younger than 75 years old showed a decreased odds of waiting for family (OR, 0.91; 95% CI: 0.89–0.94), but there were no sex difference in those over 75 years. Higher education markedly decreased the odds of waiting for the family in the 40–74 age group, but increased the same odds in the 75–99 age group. Individuals with access to more avenues to learn about stroke were better at avoiding waiting across both age groups, and this effect was stronger in the 40–74 age group (60%) than in the 75–99 age group (47%). Stroke history and family history reduced the likelihood of waiting in the 40–74 age group, but did not in the 75–99 age group. Individuals with friends afflicted by stroke were more likely to wait for family in both groups with 15% increase in the younger age group and 31% increase in the older age group.

## Discussion

Non-adjusted analysis of data from the FAST-RIGHT study shows that 34.7% of the study participants in the 40–74 age group, and 36.6% in the 75–99 age group did not call EMS first [15], even though they had considered abnormal symptoms as “stroke onset.” Similar to our results, 18.9% in Spain, [11] 28% in Sweden [22], 33.6% in America [9], and 35.5% in a small study in China [12] avoided calling an ambulance despite recognizing stroke onset. In clinical practice in China, this rate showed a significant increase to 82.1% [10]. Although several studies have reported a weak association between recognizing the onset of stroke and calling EMS, there is still a gap between knowledge and action [10–12, 23]. The main “alternative response” to stroke was to “call and wait for family, then go to hospital,” which may result in inability to receive thrombolysis [4, 5]. We propose that both the rate of intent to use EMS and the rate of receiving thrombolysis would increase remarkably if targeted interventions were carried out to change the “Wait for family” behavior to an immediate call for ambulance assistance [24].

In low– and middle– income countries including China, people aged 75 years and older had stroke incidence, prevalence, and mortality rates that were 18.8, 11.3, and 35.6 times more than those seen in people younger than 75 years, respectively [16]. Similarly, in our study, the stroke prevalence in the 75–99 age group was nearly twice as high as that in the 40–74 age group. Although the 75–99 age group was slightly more likely to call EMS after adjustment, the overall rate to call EMS did not increase significantly according to stroke risk. Hence, there is still the concern that the two age groups are not identical, and the reasons influencing their behaviors may be different. In our study, the associated socio–demographic factors were examined in the 40–74 and 75–99 age groups, separately, and significant differences were found.

Individuals in the 40–74 age group with multiple children, and those living with their family tended to wait for their family after stroke osnet. This suggests that family was a barrier to timely usage of EMS [15]. We identified several reasons for this hesitation. For example, some participants considered their family members more reliable and private transportation to be more efficient and convenient. Another concern was that they were unable to handle transactions due to lack of money and caregivers in the emergency department as the services provided are prepaid in Chinese hospitals [3]. To change their understanding of stroke onset and dispel misgivings, the “Green Channel” of emergency network for stroke should be publicized among residents in addition to education regarding the critical importance of time and the benefits of EMS usage [6].

Living alone decreased the possibility of waiting for family, but contrary to our hypothesis, this did not mean these individuals avoided staying alone at home. Previous studies showed that less than 7% patients activated EMS by themselves, and most ambulance calls were made by bystanders [24–26]. If we considered the high rate of living alone in 75–99 age group with high stroke risk, living alone was more detrimental than living with family to effectively act at the time of stroke onset. Different from those living with family, individuals living alone are required to call the EMS themselves. It is thus reasonable to educate those living alone about using EMS.

Several studies indicated that having friends previously afflicted by stroke improved individuals’ knowledge of stroke, but its effect on response to stroke onset remained unclear [27, 28]. In our study, having afflicted friends was associated with wait-and-see behaviors in both age groups. Unfortunately, further explanations cannot be provided due to limited data. The severity of stroke and prognosis of their acquaintances were not investigated and remained undetermined. It is possible that the afflicted acquaintances had minor strokes with good prognosis, leading our participants to ignore the severity and urgency of stroke onset. This unexpected funding suggests that direct stroke education is still necessary to individuals who have afflicted friends, and might underestimate the effects of a stroke.

Unexpectedly [13, 25, 29], those in the 75–99 age group with higher education levels were more likely to wait for the family than were those with lower education levels, although the reasons for this finding are unclear. We speculate that they overestimated their judgment while their medical knowledge was lacking, and hence are of even greater concern. They probably considered the elapsed time as insignificant, underestimated the severity of stroke onset, and did not know the time required for the necessary diagnostic procedures before administration of recombinant tissue plasminogen activator [3, 14].

Different for the 40–74 age group, stroke history, number of children, sex and family history did not affect the “Wait for family” behaviors in the 75–99 age group. It seems that their behavior pattern was more fixed and not susceptible to other factors. Compared with individuals in the 40–74 age group, those over 75 years’ behaviors were not influenced by number of children, which might be because their children are older and are less able to transport them to the hospital. Although multiple avenues to learn about stroke decreased the odds of waiting for the family, as shown by previous reports [30, 31], the reduction was markedly lower in the 75–99 age group. Moreover, they preferred a paper medium over the internet, despite visual impairment in over half of the population [32]. Therefore, the effect of stroke education may be limited in the 75–99 age group.

Females in the 40–74 age group tended to avoid staying at home and waiting for family when encountering stroke onset, which may be due to lower average education and fewer avenues to learn about stroke. Similarly, males in the 75–99 age group seemed performing better with small differences, though the reasons still remain complex, awaiting for exploration. However, other studies reported either no effect or controversial effect of sex on calling EMS [9, 10, 25].

There are several limitations in our study. First, closed-ended questions were to establish why residents did not call EMS after identifying stroke onset, while the underlying reasons were varied [9]. Calling a taxi, visiting general practitioners, providing first aid, or ‘something else’ were the possible choices [3, 9]. Moreover, mobility difficulties were more common in the 75–99 age group, which probably influenced their options during stroke. However, this fact was not considered in our analysis. Additionally, our non-random sampling design and selection of adults over 40 years in China from the CNSSS might affect the results and thus generalization is limited [15]. The immediate response to stroke onset might be influenced by different socio economic features, behaviors, and healthcare system in different countries [9, 11]. Finally, our study could not provide direct reasons underlying differences between the two age groups, warranting further researches.

In summary, the rate of not immediately calling EMS after recognizing stroke onset was slightly higher in the 75–99 age group than in the 40–74 age group. Although the majority of wait-and-see behaviors involved waiting for family members, the barriers of calling ambulance were different in both age groups. The behavural pattern in the 75–99 age group seemed more fixed and less susceptible to family factors. This study emphasizes the need to bridge the gap between recognition of stroke symptoms and the appropriate action [9]. Strategies should differ between both age groups, for instance, the stroke knowledge delivery may be more effective via Internet in the 40–74 age group.

## Supplementary information

**Additional file 1: Appendix 1.** List of the FAST-RIGHT Investigators and Coordinators. **Appendix 2.** The structure of FAST-RIGHT questionnaire with additional 4 questions about stroke awareness. **Appendix 3.** Definition of Risk Factors for Stroke. **Table S1.** Missing data for each variable in adults recognizing stroke. **Table S2.** Comparison of variables between 40 and 74 and 75–99 age groups. **Table S3.** Comparison of avenues between 40 and 74 and 75–99 age groups. **Table S4.** Responses to stroke among residents recognizing stroke by age groups. **Table S5.** Logistic regression models of factors associated with self-observation at home and waiting for family among residents recognizing stroke, respectively. **Table S6.** Living status in different age groups. **Table S7.** Distribution of education and number of avenues to learn about stroke in 40–74 age group categorized by sex. **Figure S1.** Data preparation and cleaning process. **Figure S2.** Prevalence of stroke and cardiovascular risk factors among 40–74 and 75–99 age groups.

## Data Availability

The data sets in this study are available from the corresponding author on reasonable request.

## Abbreviations

AF: Atrial fibrillation
CI: Confidence interval
CNSSS: China National Stroke Screening Survey
EMS: Emergency medical services
FAST-RIGHT: FAST (Fast, Arm, Speech, Time)-RIGHT (Right Response to Stroke)
OR: Odds ratio
