# Supplementary material for: Barriers from calling ambulance after recognizing stroke differed in adults younger or older than 75 years old in China
Source: BMC Neurol. 2019 Nov 12;19:283. doi: 10.1186/s12883-019-1480-6 (PMC6852842; doi:10.1186/s12883-019-1480-6)
Supplement: Supplementary file 1 — Additional file 1: Appendix 1. List of the FAST-RIGHT Investigators and Coordinators. Appendix 2. The structure of FAST-RIGHT questionnaire with additional 4 questions about stroke awareness. Appendix 3. Definition of Risk Factors for Stroke. Table S1. Missing data for each variable in adults recognizing stroke. Table S2. Comparison of variables between 40 and 74 and 75–99 age groups. Table S3. Comparison of avenues between 40 and 74 and 75–99 age groups. Table S4. Responses to stroke among residents recognizing stroke by age groups. Table S5. Logistic regression models of factors associated with self-observation at home and waiting for family among residents recognizing stroke, respectively. Table S6. Living status in different age groups. Table S7. Distribution of education and number of avenues to learn about stroke in 40–74 age group categorized by sex. Figure S1. Data preparation and cleaning process. Figure S2. Prevalence of stroke and cardiovascular risk factors among 40–74 and 75–99 age groups. [file 12883_2019_1480_MOESM1_ESM.docx]

Supplemental Material.

1. **List of the FAST-RIGHT Investigators and Coordinators**

Bin Peng, MD (PI), Shengde Li, MD, Li-Ying Cui, MD, Nan Jiang, MD, Yuehui Hong, MD, Peking Union Medical College Hospital, Department of Neurology, Beijing; Longde Wang, MD, Stroke Control Project Committee, The National Health Commission, Beijing; Craig Anderson, MD, PhD, Neurological and Mental Health Division, The George Institute for Global Health, Faculty of Medicine, University of New South Wales, Sydney, Australia; The George Institute for Global Health, Peking University Health Science Center, Beijing, China; Chengdong Yu, MD, Guangliang Shan, MD, Chinese Academy of Medical Sciences, Institute of Basic Medical Sciences, Department of Epidemiology and Statistics, Beijing; Weidong Liu, MD, Liaocheng People’s Hospital, Neurosurgical Department, Liaocheng, Shandong; Jian Li, MD, Affliated Hospital of Weifang Medical University, Neurology Department, Weifang, Shandong; Chunpeng Gao, MD, Dalian Municipal Central Hospital, Disease Control and Prevention Offce, Dalian, Liaoning; Suiqiang Zhu, MD, PhD, Huazhong University of Science and Technology, Tongji Hospital of Tongji Medical College, Department of Neurology, Wuhan, Hubei; Ping Xu, MD, Changde First People’s Hospital, Department of Neurology, Changde, Hunan; Tiemin Wei, MD, the Central Hospital of Lishui, Department of Cardiology, Lishui, Zhejiang; Yun Luo, MD, First People’s Hospital of Jiujiang, Department of Cardiovascular, Jiujiang, Jiangxi; Shengli Chen, MD, Chongqing Three Gorges Central Hospital, Department of Neurology, Chongqing; Dan Liu, MD, Jingmen First People’s Hospital, Jingmen, Hubei; Dongmei Xie, MD, Liuzhou Worker’s Hospital, Liuzhou, Guangxi; Dong Xu, Ningxia People’s Hospital, Yinchuan, Ningxia; Fei Wei, MD, Yichang Center People’s Hospital, Yichang, Hubei; Guanghui Wu, MD, Ningde City Hospital, Ningde, Fujian; Hongyan Li, MD, The People’s Hospital of Xinjiang Uygur Autonomous Region, Urumqi, Xinjiang; Hua Luo, MD, Affliated Hospital of Southwest Medical University, Luzhou, Sichuan; Jie Min, MD, The First People’s Hospital of Jingzhou, Jingzhou, Hubei; Jinhai Tang, MD, Jiangsu Province Hospital, Nanjing, Jiangsu; Jun Sun, MD, Wenzhou Central Hospital, Wenzhou, Zhejiang; Luoqing Li, MD, The First People Hospital of Yueyang, Yueyang, Hunan; Qi Yao, MD, Xinyu People’s Hospital, Xinyu, Jiangxi; Shilin Liu, MD, Pingxiang People’s Hospital, Pingxiang, Jiangxi; Wei Shi, MD, Affliated Hospital of Nantong University, Nantong, Jiangsu; Wei Yan, MD, The First People’s Hospital of Kashgar, Kashgar, Xinjiang; Xiaofei Yu, MD, Shuguang Hospital Affliated to Shanghai University of Traditional Chinese Medicine, Shanghai; Xiaopeng Luo, MD, Zhuzhou Central Hospital, Zhuzhou, Hunan; Xiaoxiang Peng, MD, Third people’s Hospital of Hubei Province, Wuhan, Hubei; Ya Zhang, MD, Dali Bai Autonomous Prefecture People’s Hospital, Dali, Yunnan; Yang Gao, MD, Yancheng City First People’s Hospital, Yancheng, Jiangsu; Ye Peng, MD, Harrison International Peace Hostipal, Hengshui, Hebei; Yongling Xue, MD, Qujing First People’s Hospital, Qujing, Yunnan; Zhi Lin, MD, Central People’s Hospital Of ZhanJiang, Zhanjiang, Guangdong; on behalf of the FAST-RIGHT study group

1. The structure of FAST-RIGHT questionnaire with additional 4 questions about stroke awareness[1]
2. **FAST-RIGHT**
3. Which disease do the following symptoms probably mean? (Facial droop, Arm weakness, Speech disturbance)

- Correct (Participant should give an answer like “stroke”, “cerebral infarct”, “Cerebral thrombosis”, “ cerebral hemorrhage”, “ cerebral embolism”, “ apoplexy”)
- Incorrect（none of above）

1. What will you do when encountering above-mentioned symptoms? (Single selection)

- Self-observation at home (incorrect)
- Call the family, wait for them, then go to hospital (incorrect)
- Call emergency phone immediately (correct)

1. Have your relatives or colleagues suffered from these diseases?:Yes/No
2. How do you get such information? (Single selection/multiple Choice)

Newspaper/TV/Broadcast/Wechat/Internet/popular science and technology (PST) activities

1. **The other relative factors in this survey: brief introduction**

- Basic Information
- Demographic information (age, sex, education, family member, marriage, career, living status, annual income, medical insurance)
- Address (province)
- The status of conducting questionnaire (by self /others/phone)
- Lifestyle
- Smoking (Yes/No/Quit/Years)
- Drinking (Yes/No/Quit/Years)
- Exercise habit (often/less frequency)
- Dietary habits
- Family history (stroke, coronary artery disease [CHD], hypertension, diabetes mellitus, dyslipidemia]
- Diseases and management during survey (2015-2017)
- cerebrovascular disease [new stroke, types, Outpatient/Inpatient, mRS ]
- Coronary Heart Disease [new CHD, atrial fibrillation [AF]/drugs]
- Hypertension [drug adherence, monitoring frequency, qualification rate]
- Diabetes Mellitus [drug adherence, monitoring frequency, qualification rate]
- Dyslipidemia [drug adherence, monitoring frequency, qualification rate]
- Physical examination (body mass index [BMI], blood pressure [BP], cardiac auscultation)
- Grade of stroke risk

Hypertension Diabetes mellitus Dyslipidemia AF

BMI-Obesity Smoking Lack of exercise

Family history History of stroke History of transient ischemic attack (TIA)

Five levels: Stroke TIA High-Risk Moderate-Risk Low-Risk

- EEG, serum test (glucose, lipid, homocysteine), carotid artery ultrasound for new and prior high-risk, stroke and TIA group.
- Surgery and interventional therapy during this survey
- Carotid artery [CAS, CEA, Extracranial-Intracranial Bypass]
- Coronary artery [PCI, CABG]
- Surgery/Interventional therapy for Intracranial hemorrhage [Yes/No]

1. Definition of Risk Factors for Stroke[1]

| **Risk factor** | **Criteria** |
| --- | --- |
| Smoking | ≥6 months in life (accumulative or consecutive) |
| Alcohol drinking | ≥100mL spirit alcohol >3 times per week (self report) |
| Hypertension | 1. Systolic BP ≥140mmHg or diastolic BP ≥90mmHg, or taking BP-lowering drugs   OR   1. ABPM: 24 h mean BP ≥130/80 mmHg, or daytime BP ≥135/85mmHg, or nocturnal BP ≥120/70mmHg   OR  3. Home BP monitoring ≥135/85 mmHg(self report) |
| Dyslipidemia | LDL-C ≥1.8mmol/L, or HDL-C <1.04mmol/L, or TC ≥6.22mmol/L, or TG≥2.26mmol/L |
| Diabetes | 1. Fasting glucose ≥7.0 mmol/L or non-fasting blood glucose ≥11.0mmol/L with diabetes mellitus symptoms   OR  2. Fasting glucose ≥7.0mmol/L or non-fasting blood glucose ≥11.0mmol/L more than two times without typical DM symptoms  OR  3 .OGTT (75g glucose): 2 h blood glucose ≥11.0 mmol/L  OR   1. Taking glucose-lowering drugs |
| Significant overweight or obese | BMI ≥26.0 kg/m^2^ |
| AF | Either a history of persistent AF or supported by past ECG or ECG examination in this survey and confirmed by a cardiologist |
| Physical exercise | 1. ≥ 30 min of medium strength and above exercise every time, >3 times per week   OR  2. Moderate or heavy manual workers |
| Family history of stroke | Any parent or sibling with stroke; further inquiry and confirmation by a neurologist |
| History of stroke. | Either neurological deficit symptom at onset or symptomatic lacunar cerebral infarction on imaging, confirmed by a neurologist |
| TIA | Sudden onset focal/global neurological deficit lasting less than 24h, usually alleviating within 30minutes, excluding non-angiogenesis, and confirmed by a neurologist |

Table S1. Missing data for each variable in adults recognizing stroke

| Subgroup | 40–74 Age Group | 75–99 Age Group |
| --- | --- | --- |
| Age^a^ | 24 | |
| Sex | 0 | 0 |
| Site | 0 | 0 |
| Region | 0 | 0 |
| BMI^b^ | 197 | 24 |
| Education | 2 | 3 |
| Annual personal income | 40 | 9 |
| Living status^c^ | 423 | 69 |
| Children number | 213 | 23 |
| Stroke amongst people around them | 1 | 0 |
| Number of avenues to learn about stroke | 25 | 4 |
| Smoking status | 13 | 3 |
| Level of exercise | 13 | 3 |
| Family history of stroke | 119 | 11 |
| History of cerebral vascular disease | 72 | 9 |
| History of heart disease | 73 | 7 |
| History of hypertension | 77 | 8 |
| History of diabetes | 89 | 8 |
| History of dyslipidemia | 84 | 8 |

^a^ Age ≥100 was classified as missing

^b^ BMI >50 or <10 was classified as missing

^c^ Those with other type of living status were classified as missing

**Table S2. Comparison of variables between 40**–**74 and 75**–**99 age groups**

|  | **Case, N (%)** | |  |
| --- | --- | --- | --- |
| **Variables** | **40**–**74 Age Group** | **75**–**99 Age Group** | **P value** |
| **Sex** |  |  | 0.0476 |
| Male | 60270(45.4) | 9691(46.2) |  |
| Female | 72389(54.6) | 11301(53.8) |  |
| **Sites** |  |  | 0.0801 |
| Urban | 68291(51.5) | 10670(50.8) |  |
| Rural | 64368(48.5) | 10322(49.2) |  |
| **Regions** |  |  | <0.0001 |
| North + Northeast | 10209(7.7) | 940(4.5) |  |
| East | 41383(31.2) | 5574(26.5) |  |
| Central | 41284(31.1) | 7282(34.7) |  |
| South | 13576(10.2) | 3473(16.5) |  |
| Southwest | 14664(11.1) | 2155(10.3) |  |
| Northwest | 11543(8.7) | 1568(7.5) |  |
| **Education** |  |  | <0.0001 |
| ≤ Primary | 51543(38.8) | 13755(65.5) |  |
| Middle/High school | 72004(54.3) | 6274(29.9) |  |
| ≥ College | 9110(6.9) | 960(4.6) |  |
| **Personal Annual Income, US $** |  |  | <0.0001 |
| < 731 | 32183(24.3) | 8093(38.6) |  |
| 731–2923 | 46103(34.7) | 6261(29.8) |  |
| > 2923 | 54333(41.0) | 6629(31.6) |  |
| **Living Status^a^** |  |  | <0.0001 |
| Living alone | 2934 (2.2) | 1422 (6.8) |  |
| With spouse | 122418 (92.6) | 16158 (77.2) |  |
| With others | 6884 (5.2) | 3343 (16.0) |  |
| **Children number** |  |  | <0.0001 |
| 0 | 1114(0.9) | 112(0.5) |  |
| 1 | 41906(31.6) | 1752(8.4) |  |
| 2–3 | 79248(59.8) | 12467(59.4) |  |
| ≥ 4 | 10178(7.7) | 6638(31.7) |  |
| **Stroke in others^b^** |  |  | 0.1107 |
| No | 106709(80.4) | 16787(80.0) |  |
| Yes | 25949(19.6) | 4205(20.0) |  |
| **Avenues ^c^** |  |  | <0.0001 |
| 1 | 61836(46.6) | 9902(47.2) |  |
| 2–3 | 62893(47.4) | 10394(49.5) |  |
| 4–6 | 7905(6.0) | 692(3.3) |  |
| **Family history of stroke** |  |  | <0.0001 |
| No | 116008(87.5) | 18132(86.4) |  |
| Yes | 9423(7.1) | 988(4.7) |  |
| Unknown | 7109(5.4) | 1861(8.9) |  |

^a^ With spouse includes living with spouse or both spouse and children; With others includes living with children, living in nursing home, and with other people. Those with other type of living status were classified as missing data.

^b^ Relatives or colleagues who have suffered an acute stroke

^c^ Number of avenues taken to learn about acute stroke

**Table S3. Comparison of avenues between 40–74 and 75–99 age groups**

|  | 40–74 Age Group | 75–99 Age Group | P value |
| --- | --- | --- | --- |
|  | N (%) | N (%) |  |
| Newspaper | 35061(26.4) | 6303(30.0) | <0.0001 |
| Television | 104811(79.0) | 16499(78.6) | 0.1745 |
| Broadcast | 22694(17.1) | 3650(17.4) | 0.3162 |
| Wechat | 13177(9.9) | 904(4.3) | <0.0001 |
| Internet | 11168(8.4) | 740(3.5) | <0.0001 |
| PST activities^a^ | 56754(42.8) | 9108(43.4) | 0.0991 |

^a^ PST denotes popular science and technology

**Table S4. Responses to stroke among residents recognizing stroke by age groups**

|  |  | Responses to stroke, N (%) | | |  |
| --- | --- | --- | --- | --- | --- |
| Age, y | Case, N (%) | Self-observation  at home | Wait for family^a^ | Call EMS | P Value |
| 40-74 Age Group | 132659(86.3) | 3912(3.0) | 42071(31.7) | 86676(65.3) | <0.0001 |
| 75-99 Age Group | 20992(13.7) | 738(3.5) | 6957(33.1) | 13297(63.4) |  |

^a^ Call family member, wait for them, and then go to hospital

**Table S5. Logistic regression models of factors associated with self-observation at home and waiting for family among residents recognizing stroke, respectively**

|  | **OR (95%CI)** | |
| --- | --- | --- |
| **Associated factors** | **Self-observation at home** | **Wait for family** |
| **75–99 years (Ref: 40–74 years)** | 0.88 (0.81–0.97) | 0.88 (0.85–0.91) |
| **Female (Ref: Male)** | 0.91 (0.86–0.97) | 0.92 (0.90–0.94) |
| **Rural (Ref: Urban)** | 0.84 (0.78–0.90) | 1.58 (1.54–1.62) |
| **Education (Ref:≤Primary)** |  |  |
| Middle/High school | 1.15 (1.07–1.24) | 0.86 (0.84–0.88) |
| ≥College | 0.97 (0.85–1.12) | 0.96 (0.91–1.01) |
| **Personal annual income (US $) (Ref: < 731 )** |  |  |
| 731–2923 | 0.85 (0.78–0.92) | 0.67 (0.65–0.69) |
| >2923 | 0.86 (0.79–0.94) | 0.53 (0.51–0.55) |
| **Living status^a^ (Ref: With spouses)** |  |  |
| Alone | 0.96 (0.79–1.16) | 0.75 (0.70–0.80) |
| With others | 1.27 (1.14–1.41) | 0.97 (0.92–1.01) |
| **Children number (Ref: 0)** |  |  |
| 1 | 1.04 (0.72–1.52) | 0.98 (0.85–1.13) |
| 2–3 | 0.82 (0.57–1.19) | 1.39 (1.21–1.59) |
| ≥4 | 1.85 (1.27–2.69) | 1.46 (1.27–1.68) |
| **Stroke in others^b^ (Ref: No)** | 1.62 (1.51–1.73) | 1.19 (1.15–1.22) |
| **Avenues^c^ (Ref: 1)** |  |  |
| 2–3 | 0.91 (0.86–0.97) | 0.61 (0.59–0.62) |
| 4–6 | 0.27 (0.22–0.33) | 0.41 (0.39–0.44) |
| **Family history of stroke (Ref: No)** |  |  |
| Yes | 0.56 (0.48–0.65) | 0.87 (0.83–0.92) |
| Unknown | 3.70 (3.38–4.04) | 1.54 (1.47–1.62) |
| **History of CVD^d^ (Ref: No)** | 0.51 (0.41–0.62) | 0.85 (0.80–0.91) |

This logistic model was adjusted by regions.

^a^ With spouse includes living with spouse or both spouse and children; With others includes living with children, living in nursing home, and with other people. Those with other type of living status were classified as missing data.

^b^ Relatives or colleagues who have suffered an acute stroke

^c^ Number of avenues taken to learn about acute stroke

^d^ CVD denotes cerebral vascular disease, including ischemic stroke, transient ischemic anemia, cerebral hemorrhage, and subarachnoid hemorrhage.

**Table S6. Living status in different age groups**

|  | **Case, N (%)** | | | | |  |
| --- | --- | --- | --- | --- | --- | --- |
|  | 40–49 years | 50–59 years | 60–69 years | 70–79 years | 80–99 years | P value |
| Living alone | 433 (1.3) | 650 (1.5) | 1174 (2.8) | 1307 (5.1) | 792 (7.7) | <0.0001 |
| Living with spouses | 31284 (94.9) | 39836 (94.2) | 38511 (91.3) | 21577 (84.8) | 7368 (71.9) |  |
| Living with others | 1262 (3.8) | 1811 (4.3) | 2495 (5.9) | 2566 (10.1) | 2093 (20.4) |  |

Living status: With spouse includes living with spouse or both spouse and children; With others includes living with children, living in nursing home, and with other people. Those with other type of living status were classified as missing data.

**Table S7. Distribution of education and number of avenues to learn about stroke in 40**–**74 age group categorized by sex**

|  | **Sex, N (%)** | |  |
| --- | --- | --- | --- |
|  | Male | Female | P value |
| Education ^a^ |  |  | <0.0001 |
| ≤ Primary | 20764 (34.5) | 30779 (42.5) |  |
| Middle/High school | 34542 (57.3) | 37462 (51.8) |  |
| ≥ College | 4964 (8.2) | 4146 (5.7) |  |
| Avenues ^b^ |  |  | <0.0001 |
| 1 | 27715 (46.0) | 34121 (47.1) |  |
| 2–3 | 28663 (47.6) | 34230 (47.3) |  |
| 4–6 | 3878 (6.4) | 4027 (5.6) |  |

a Missing data:2

b Number of avenues taken to learn about acute stroke; missing data:25.

**Figure S1.** **Data preparation and cleaning process[1]**

Delete: 2437

**Raw data in primary survey (N=243 279)**

Delete: 51126

**Data according to age standard (N=240 842)**

**Data of finishing face-to-face interview by oneself (N=189 716)**

**Final dataset (N=187 723)**

Delete: 1993

Step7: Did not answer question of stroke recognition (n= 1991)

Step8: Did not answer question of response to stroke (n= 2)

Step1: Death (n= 2413)

Step2: Test population^a^ (n=5)

Step3: Age < 40 years (n=13) and age ≥ 146 year (n=6)

Step4: Loss of follow-up (n= 6338)

Step5: Finish interview by others (n= 7240)

Step6: Finish interview via phone^b^ (n= 37548)

Recognize stroke: 153 675

Not recognize stroke: 34 048

^a^ Virtual population to test the data management system.

^b^ To ensure the accuracy, integrity, and creditability, residents finishing questionnaires via phone in CNSSS was not included in FAST-RIGHT study.

**Figure S2. Prevalence of stroke and cardiovascular risk factors among 40**–**74 and 75**–**99 age groups**

**

**

Overweight and Obesity indicates BMI between 24 and 50.

Smoking includes current, former, and passive smoking.

History of cerebral vascular disease includes ischemic stroke, transient ischemic anemia, cerebral hemorrhage, and subarachnoid hemorrhage.

**Reference**

1. Li S, Cui LY, Anderson C, Zhu S, Xu P, Wei T, et al. Public Awareness of Stroke and the Appropriate Responses in China: A Cross-Sectional Community-Based Study (FAST-RIGHT). Stroke. 2019;50:455-462. DOI: 10.1161/STROKEAHA.118.023317.
